# Abrupt Withdrawal From Chronic High-Dose Zolpidem Use: A Case Report of Resulting Delirium

**DOI:** 10.7759/cureus.49025

**Published:** 2023-11-18

**Authors:** Harshal Awasthi, Abhimanyu Vohra

**Affiliations:** 1 Psychiatry, Matra Chhaya Medical Center, Kanpur, IND; 2 Psychiatry, Interior Health, Cranbrook, CAN; 3 General Medicine, Matra Chhaya Medical Center, Kanpur, IND

**Keywords:** withdrawal, acute delirium, insomnia, zolpidem, case report, patient education, delirium, addiction, unguided dosage modification, zolpidem abuse or misuse

## Abstract

We report the case of a male in his twenties who was prescribed 10 mg of zolpidem daily for sleep disturbances. Within one month, he self-augmented the dose to 30 mg daily. Unable to secure an authorized refill, he sought unauthorized suppliers and increased his daily intake to 70 mg over eight months. One day after his medication supply was depleted, he presented to the emergency department with symptoms indicative of acute delirium. Delirium was successfully alleviated within six hours using lorazepam. This was followed by a five-day lorazepam tapering regimen during the patient's hospital stay and then a subsequent four-day taper in an outpatient setting. This case highlights the dangers associated with zolpidem misuse, the swift onset of withdrawal symptoms following abrupt discontinuation, and the crucial importance of rigorous prescription monitoring and patient education regarding the risks of unguided dosage modifications and the sudden cessation of zolpidem.

## Introduction

Zolpidem, a non-benzodiazepine sedative-hypnotic, is approved for the short-term management of insomnia [1]. Initially regarded as a safer alternative to benzodiazepines [2-5], its reputation has been challenged owing to increasing reports of misuse. While adverse outcomes from zolpidem are well-documented, instances of severe withdrawal symptoms, such as delirium in young individuals without prior substance abuse or psychiatric histories, are rare. Herein, we report a unique case of a young male in his twenties who experienced acute delirium following the abrupt discontinuation of high-dose zolpidem abuse. This case underscores the imperative need to remain vigilant about zolpidem dependence in young individuals. It further highlights the perils of unsupervised dose escalation and the swift emergence of withdrawal symptoms upon abrupt discontinuation, despite the absence of concurrent medical or psychiatric comorbidities. It emphasizes the need for vigilant prescription oversight and patient education in highlighting the potential risks associated with unauthorized dosage adjustments and abrupt discontinuation of medication.

## Case presentation

A male in his mid-20s was admitted to the emergency department exhibiting symptoms of agitation, confusion, and perceptual disturbances, including visual hallucinations. Owing to the patient's presenting condition, we procured initial clinical history through collateral information from his live-in partner, who was deemed a reliable informant.

Ten months earlier, he had been prescribed zolpidem (10 mg daily) to manage sleep disturbances associated with occupational stress. Within a month, he unilaterally increased the dosage to 30 mg a day without consulting a physician. As he struggled to obtain authorized refills, he resorted to illicit street supply, raising his dosage to 70 mg a day for over eight months. His partner reported that he had appeared to be "zoned out" over the past few months but had mostly remained functional.

According to the patient's partner, the patient experienced difficulties in reaching his supplier the previous afternoon, which led to increased anxiety, profuse sweating, and, consequently, insomnia. On the day of the occurrence, the patient’s partner left for work in the morning, and, upon her return, approximately an hour before the clinical assessment, she discovered the patient pacing the hallway, heavily perspiring, speaking incoherently, and apparently interacting with nonexistent entities.

The patient had no prior history of similar episodes. His medical records indicated controlled asthma, managed with a salmeterol + fluticasone inhaler (25/250 mcg), and no other medications. His psychiatric history was limited to stress-induced insomnia, which had been managed with zolpidem. There was no known history of substance use, except for occasional social alcohol consumption. The patient’s history was unremarkable, and in terms of his social life, he was a young male pursuing a college degree while working part-time. He lived with his partner in a rented apartment. Furthermore, no recent stressors were identified upon admission to the emergency department.

Upon examination, the patient’s vital signs were normal, except for an elevated heart rate of 104 beats/min. A physical examination revealed no focal neurological deficits or external injuries, and all other findings were within normal limits. A thorough mental status examination was not feasible because of the patient's limited cooperation. However, clinical observations indicated intermittent, profound confusion and disorientation regarding time, place, and person. Moments of lucidity were fleeting and lasted only a few seconds. Additionally, the patient was uncooperative and exhibited confrontational behavior. He reported seeing individuals in the room and engaging in conversations with them, indicative of visual hallucinations.

Routine blood tests, including a complete blood count, serum biochemistry, and electrolyte tests, all yielded normal results. Blood glucose analysis ruled out hypo- or hyperglycemia. The urine drug toxicology screen tested negative for the five drug classes examined: amphetamines, benzodiazepines, cannabinoids, cocaine, and opiates. Specific drug tests for zolpidem were not available at the facility. Further diagnostic assessments, including electrocardiography, computed tomography, and electroencephalography, did not reveal any abnormalities (see Table 1).

**Table 1:** Investigation Results

Test	Results
CBC (complete blood count)	WBC: 6.7 x 10^3/μL, RBC: 4.5 x 10^6/μL, Hb: 13.5 g/dL, platelets: 200 x 10^3/μL
Serum biochemistry (typical tests)	Creatinine: 0.8 mg/dL, total protein: 7.0 g/dL, albumin: 4.0 g/dL, bilirubin: 0.6 mg/dL
Electrolyte levels	Sodium: 140 mEq/L, potassium: 4.2 mEq/L, calcium: 9.0 mg/dL
Blood glucose levels	Fasting blood glucose: 90 mg/dL
Urine drug toxicology	Negative for amphetamines, benzodiazepines, cannabinoids, cocaine, and opiates
Zolpidem-specific test	Not available at the facility
ECG (electrocardiogram)	No abnormalities detected
CT scans	No significant findings
EEG (electroencephalogram)	No epileptiform activity observed

Given the clinical assessment and information provided, treatment for zolpidem withdrawal was initiated using lorazepam. The delirium symptoms resolved within six hours. The patient's lorazepam was then tapered over a five-day hospital stay, followed by a four-day outpatient taper in the community setting.

During the four-week outpatient clinic follow-up, the patient reported abstinence from zolpidem and exhibited a normal mental status. While he continued experiencing intermittent sleep disturbances, he declined further psychiatric evaluations and sleep study referrals. Subsequent attempts to follow up with the patient were unsuccessful.

## Discussion

Zolpidem, a non-benzodiazepine hypnotic approved by the Food and Drug Administration (FDA), is an established therapeutic modality for short-term insomnia, particularly in facilitating sleep initiation [2]. Its unique imidazopyridine structural configuration suggests a specific affinity for the α1 subunit of the gamma amino butyric acid A (GABA) receptor, indicating its possible role in modulating the GABA-benzodiazepine receptor complex [3,4].

The introduction of non-benzodiazepine hypnotics was met with optimism because of their perceived enhanced safety profile compared to conventional benzodiazepines [5-7]. However, emerging evidence has necessitated a reevaluation of their pharmacodynamic and safety profiles.

Recent literature provides insight into a concerning trend in the misuse of hypnotics, especially zolpidem [7-9]. Increasing prescription rates for zolpidem have coincided with a rise in hospital admissions related to its acute side effects with clinical presentations spanning from psychotic episodes and visual hallucinations to parasomnias [10,11]. This observation is especially concerning considering the FDA's advisory in 2013, which aimed at curtailing such events [12].

Furthermore, the challenges associated with zolpidem discontinuation, particularly after extended high-dose use, are drawing increased attention. Numerous studies underscore the manifestation of severe withdrawal symptoms including seizures after abrupt zolpidem cessation [13,14].

While documented instances of delirium resulting from zolpidem withdrawal exist, they are relatively rare in the literature. Most of these cases have involved older patients or those with histories of alcohol abuse or psychiatric conditions. Aggarwal and Sharma documented zolpidem withdrawal delirium in 2010 [15]. Chien et al. further elaborated on symptoms like delirium, seizure, and acute psychosis in 2011 [16]. Innamuri et al. discussed the management complexities of zolpidem dependence in 2023 [17]. Chopra et al. highlighted the amplified adverse effects of zolpidem because of concurrent alcohol and SSRI use [18]. Mattoo, Gaur, and Das addressed zolpidem withdrawal delirium in 2011 [19], and Chate et al. reported on zolpidem-induced delirium in 2013 [20].

Our study, however, presents a distinct scenario, highlighting acute delirium in a young individual devoid of such risk factors, which emphasizes the necessity of a broader understanding of zolpidem's potential side effects. This pivotal case serves as a significant addition to the current literature, spotlighting the vulnerability of young, ostensibly healthy individuals to acute delirium after sudden zolpidem withdrawal. It underscores the latent risks of zolpidem misuse, even in those seemingly detached from the standard risk parameters. This study, when considered alongside prior findings, accentuates the pressing need for meticulous prescription surveillance and a comprehensive approach to patient guidance. Furthermore, it underscores the essential role that healthcare professionals play in recognizing and mitigating the grave consequences of unauthorized high-dose zolpidem use and the perils of its abrupt cessation.

## Conclusions

The findings of this case highlight the importance of exercising caution when prescribing zolpidem. It is essential for healthcare providers to approach the prescription of zolpidem with a level of scrutiny similar to that applied to benzodiazepines, especially in light of the growing body of evidence regarding its potential for addiction.

Abrupt cessation of zolpidem, particularly at higher doses, demands vigilance for potential withdrawal symptoms, similar to those observed with the discontinuation of benzodiazepines. Given the widespread misuse of zolpidem and its absence from routine emergency room drug screens, it is imperative for emergency medical personnel to be able to recognize potential zolpidem withdrawal symptoms. Furthermore, patient education plays a pivotal role in mitigating the risks associated with unsupervised use and dose escalation. It is essential to inform patients about the potential consequences of such practices and to encourage open communication regarding their medication use, particularly in cases involving non-benzodiazepine hypnotics like zolpidem.
